# The Meaning of Sedentary Behavior as Experienced by People in the Transition From Working Life to Retirement: An Empirical Phenomenological Study

**DOI:** 10.1093/ptj/pzab117

**Published:** 2021-05-05

**Authors:** Caroline Eklund, Magnus L Elfström, Petra von Heideken Wågert, Anne Söderlund, Catharina Gustavsson, Sara Cederbom, Charlotta Thunborg, Helena Lööf

**Affiliations:** Division of Physiotherapy, School of Health, Care and Social Welfare, Mälardalen University, Västerås, Sweden; Division of Psychology, School of Health, Care and Social Welfare, Mälardalen University, Eskilstuna, Sweden; Division of Physiotherapy, School of Health, Care and Social Welfare, Mälardalen University, Västerås, Sweden; Division of Physiotherapy, School of Health, Care and Social Welfare, Mälardalen University, Västerås, Sweden; Center for Clinical Research Dalarna, Uppsala University, Falun, Sweden; School of Education, Health and Social Studies, Dalarna University, Falun, Sweden; Department of Public Health and Caring Sciences, Uppsala University, Uppsala, Sweden; Centre for Development, Social Welfare and Care, Västmanland County Council, Västerås, Sweden; Department of Neurobiology, Care Sciences and Society, Division of Clinical Geriatrics, Center for Alzheimer Research, Karolinska Institute, Stockholm, Sweden; Division of Caring Science, School of Health, Care and Social Welfare, Mälardalen University, Västerås, Sweden; Department of Health Promoting Science, Sophiahemmet University, Stockholm, Sweden

**Keywords:** Healthy Aging, Lifestyle, Phenomenology, Physical Activity, Sedentary

## Abstract

**Objective:**

Sedentary behavior (SB) is defined as a mean of >6 hours of daytime sitting or lying down. SB has been shown to increase with older age and is a risk factor for disease. During the transition from working life to retirement, changes in daily life activities occur, risking increased SB. The aim of the present study was to gain a deeper understanding of SB in relation to the transition from working life to retirement as experienced by persons in retirement.

**Methods:**

The study was grounded in a phenomenological life-world perspective. Fourteen semi-structured interviews were conducted with participants aged 64 to 75 years. Data were analyzed using the empirical phenomenological psychological method.

**Results:**

The participants described that voluntary sedentary time was positively related to general health and well-being, whereas involuntary sedentary time was negatively related to health. Increased sedentary time was described as natural when aging. Retirement was expressed as a time for rest after hard work and the ability to choose a slower pace in life. Internal and external demands and daily routines interrupted SB, whereas loneliness was perceived to increase SB. Participants strived to find a balance between physical activity and sedentary time. The variations in the participants’ descriptions formed 3 typologies: in light of meaningful SB, in the shadow of involuntary SB, and a dual process—postponing SB with physical activity.

**Conclusion:**

Increased SB was perceived as natural when aging but something that may be postponed by conscious choices. SB was perceived as associated with health, rest, and recovery but also with the risk of deteriorating health.

**Impact:**

This knowledge of the experienced meaning of SB could guide the design of health promotion interventions and may be helpful in targeting those in need of support and individualizing interventions to decrease SB in retirement.

**Lay Summary:**

This study reveals how persons in retirement describe sedentary behavior as something healthy but also as unhealthy and that sedentary behavior is natural in aging and can be postponed by physical activity.

## Introduction

The population of older adults is increasing worldwide.^1^ The physiological changes the aging body undergoes contribute to an increased risk for many diseases and also a decline in general physical capacity, leading to the aging body being more vulnerable to health problems.^1^ This may contribute to ill health and a heavier load on health care systems.

Sedentary behavior (SB) is defined as a mean of >6 hours of sitting or lying down daily, excluding during the nighttime. SB is also defined as energy expenditure <1.5 metabolic equivalent.^2^^,^^3^ Metabolic equivalent is a ratio of working metabolic rate relative to resting metabolic rate, and this definition is encouraged to use by the Sedentary Behavior Research Network because it is more precise for estimating SB compared with hours reported.^2^ Physical inactivity can be defined as less than 150 minutes of moderate-intensity aerobic physical activity or less than 75 minutes of vigorous-intensity aerobic physical activity throughout the week and less than 2 weekly bouts of muscle-strengthening activities.^4^^,^^5^ A person can thus have SB but still be physically active. SB has been shown to increase with older age^6^ and is highly prevalent in this group.^2^^,^^7^^,^^8^ SB is also associated with ill health, and in a large meta-analysis, it was concluded that those who sat >4 h/d and had a low physical activity level had an increased risk of dying earlier.^9^

An earlier study indicated that recently retired older adults might be unfamiliar with the concept of SB or its negative health implications.^10^ Moreover, a number of factors might contribute to SB: physical limitations^11^ (eg, perceived pain^12^) as well as psychosocial factors, such as a lack of motivation,^11^ a lack of energy, social pressure to rest in older age, agist stereotyping,^12^ and environmental factors^12^ (eg, availability of community resources,^11^^,^^13^ physical infrastructure^13^ such as access to attractive facilities for physical activity,^14^ and cultural differences^11^). Older adults may see their bodies as vulnerable and feel that they, as older adults, are allowed a sedentary lifestyle.^15^ For many older adults, engagement in non-SB through structured physical activity is not appealing.^16^^,^^17^ This is often related to a reluctance to exercise because they do not regard themselves as “sporty.”^17^ Enjoyment, socialization, and a sense of achievement are crucial for older adults to take part in non-sedentary activities.^15^ Additionally, being physically active before retirement might also contribute to continued regular physical activity in retirment.^15^ However, shortcomings of previous studies are lack of analytical transparency,^12^ a focus on socially active persons,^11^ and a focus on needs regarding physical activity rather than on SB.^10^

It is important to understand older adults’ perspective on SB because interventions aiming at promoting physical activity and decreasing SB are not informed by older adults’ perspective and might therefore be perceived as less purposeful among the older adults,^15^ leading to low adherence. Understanding the meaning of SB in persons transitioning from working life to retirement can guide the design of health promotion interventions. Therefore, the aim was to gain a deeper understanding of SB in relation to the transition from working life to retirement, as experienced by persons in retirement.

## Methods

### Design

This study was grounded in an empirical phenomenological life-world perspective.^18–20^ Phenomenology^18^^,^^20^ focuses on the meaning and significance of experiences. Taking departure from the life-world perspective, the phenomenon is inseparably connected to the individual who experiences it, and the description is from a first-person point of view; no experience can be dismissed as false.

Husserl^18^ stated that people share perceptions of a phenomenon and that this *intersubjectivity* is essential for the living world (ie, the world that we share), and a phenomenon can have common “denominators” for different people.^18^ The use of phenomenological research designs to deepen the understanding of different phenomena, such as lived experience, has provided important perspectives in physical therapy.^21^ The literature reflects 2 different approaches in phenomenology; the hermeneutical and the empirical. In this study, a deep understanding and meaningful insight from the lived experience among the participants was aimed for, and therefore the empirical approach was chosen.

### Participant Recruitment

A purposive sampling technique was used (ie, snowball sampling). The inclusion criteria were retired community-dwelling older adults aged 60 to 75 years who were able to speak and understand the Swedish language and read and comprehend the study instructions. Exclusion criteria were self-reported serious disease (eg, diagnosed dementia, severe musculoskeletal disease that would hinder the participants being physically active such as hemiplegia, chronic heart failure, severe COPD, or severe depression), severe loss of vision or communicative ability, and/or working 8 h/wk or more.

In Sweden, retirement organizations are non-governmental organizations consisting of retired persons who look after the interests of persons in retirement. They also provide a platform for social events. A regional retirement organization in the middle of Sweden was visited by the first author (C.E.), who presented the study to potential participants. Interested persons were prompted to contact the first author for more information if interested to participate. One participant contacted the research team after reading about the study in a local newspaper. No one declined participation in the study after receiving personal information about the study from the researchers.

The final sample comprised 14 participants (Table 1), representing a variety of sociodemographic and socioeconomic aspects, SB, and living conditions. Eight persons were recruited after being referred by another participant. One of the persons recruited for pilot interviews was also included in the study. After participants were verified to satisfy the inclusion and exclusion criteria, all participants received oral and written information regarding the study according to ethical guidelines, including their rights, and provided written informed consent before the interview started.

**Table 1:** Participants’ Characteristics

**Participant Pseudonym**	**Age (y)**	**Sex**	**Self-Reported Sedentary Time Per Day (h)**	**Living Conditions**	**Former/Last Occupation as Physically Active/Sedentary**	**Civil Status**	**Age When Retired (y)**	**Recruitment**
John	71	M	6–7	House	Active	Married	65	Pilot study
Karen	75	F	6 (very unsure)	Apartment	Sedentary	Married	65	Retirement organization
Peter	75	M	8–10 (differs winter–summer)	Apartment	Sedentary	Married	65	Retirement organization
Henry	68	M	8	House	Sedentary	Alone	67	Snowball
Mary	68	F	15	Apartment	Sedentary	Alone	55	Newspaper
Sylvia	71	F	5–6	Apartment/ summer house	Sedentary	Alone	67	Snowball
Anna	67	F	5–6 (unsure)	House	Sedentary/active	Married	63	Snowball
Paul	68	M	6 (8 during winter)	House	Sedentary	Married	62	Snowball
Judy	72	F	8	Apartment	Sedentary/active	Married	65	Snowball
Walter	72	M	3–4	Apartment	Sedentary/active	Married	60	Snowball
Kenneth	74	M	3–4	House	Active	Married	63	Retirement organization
Cheryl	72	F	4	House	Sedentary/active	Married	62	Snowball
Carole	68	F	10	Apartment	Sedentary	Married	58	Snowball
Barbara	64	F	6	Apartment	Active	Alone	64	Snowball

### Pilot Study

The interview guide was tested to determine whether it was possible to address the questions among both health care professionals (n = 4) and potential participants (n = 2), which constituted a preliminary study according to Aspers.^22^

Pilot testing among potential participants were conducted by the first author (C.E.) and among health care professionals by the last author (H.L.). The health care professionals were 2 females, 1 registered nurse and 1 registered physical therapist, and 2 male medical doctors working within rheumatology and medicine units. The number of years of professional experience ranged from 10 to 40 years. The potential participants were persons who fit the study inclusion and exclusion criteria and were chosen by the first author. One man and 1 woman representing different occupations, both active and sedentary, were recruited. Health care professionals were acquaintances of the last author.

The interview guide was confirmed to have a high level of usefulness. Questions 1 and 4.1 were added during the pilot interviews and to the final interview guide. One of the participants in the pilot study agreed to be included in the final sample. After the participant signed informed consent, the interview was thus included in the analysis. The interview questions were not revised after that interview.

### Research Team

All authors except the second and the last author are physical therapists. The second author is a licensed clinical psychologist, and the last author is a registered nurse. All authors have a PhD. The physical therapists have different clinical backgrounds and research areas such as pain management, geriatrics/gerontology, physical activity, health promotion, behavioral medicine, and lifestyle-related behaviors. The clinical background of all authors is varied: geriatrics, rheumatology, respiration, musculoskeletal, neurology, pain, and more. Years of research experience varies from 8 to 25 years within the author group. All authors have experience with qualitative methods and interview methodology. The first author has experience in interview methodology from research and also clinical contexts. The last author has performed several studies with a phenomenological approach using interviews.

### Data Collection

**Figure 1:**
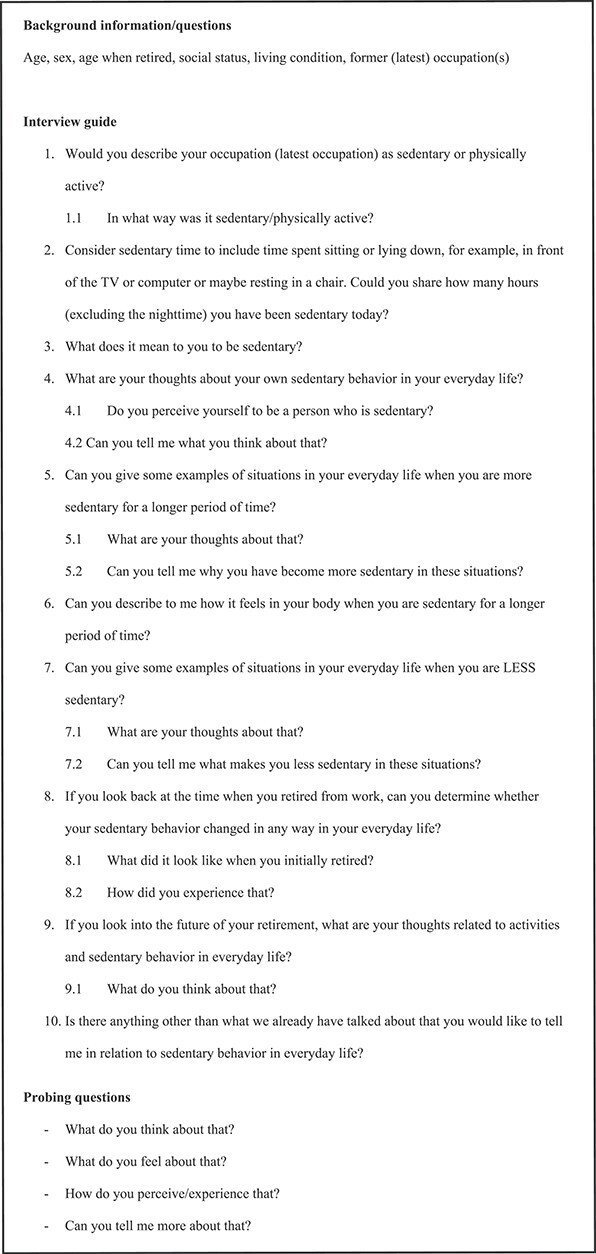
Interview guide.

Data were collected through individual in-depth face-to-face interviews using a semi-structured interview guide focusing on SB (Fig. 1). The participants were asked to select a comfortable place for the interview, and the selection of the location was determined jointly after that. All interviews were conducted in central Sweden at the participant’s home except 2, where the participants preferred to come to the university. The interview questions were designed to stimulate narratives and to attain representation of the diversity of lived experiences of the phenomenon. The interviews aimed to investigate what being sedentary means from the participant’s perspective by prompting the participants to describe situations in daily life in relation to sedentary time and behavior. Interviews were performed by the first author. All participants were unfamiliar with the first author performing the interviews except for the person recruited for the pilot interview and included in the present study as well as 1 participant who had worked within the same organization as the first author 10 years earlier.

The interviews were performed over 6 months during 2019. The interviews lasted from 24 to 54 minutes (mean, 36 minutes). Field notes were taken regarding the atmosphere during the interviews as well as for time, date, and location of the interviews. After 10 interviews, the descriptions of the phenomena in focus started to be repeated, and no new descriptions were added during the last 4 interviews. This was also ensured during the analysis process because every interview constituted 1 synopsis and thus 1 unit.

### Data Analysis

The interviews were audio-recorded and transcribed verbatim. Transcriptions were performed by the first author. Data were interpreted by using the empirical phenomenological psychological (EPP) method,^23^ which is a combined phenomenological and hermeneutical method. EPP was chosen as a method for analyzing data to learn from the informants’ experiences about SB. The analysis followed the 5 steps (Tab. 2) described by Karlsson.^23^ The first (C.E.) and last (H.L.) author performed the analysis.

**Table 2:** The Five Steps of the Empirical Phenomenological Psychological Method as Described by Karlsson^23^ (1993) and Definition of Epoché

**Analysis Steps**	**Content**
1) Getting familiar with data	Interviews were listened to and transcribed Transcribed interviews were read through several times to grasp the whole
2) Meaning units	Meaning units (MUs) were identified and researchers searched for shifts in meaning regarding phenomenon in focus of present study
3) Eidetic induction	Eidetic interpretation of MUs was performed based on everyday language used by participants as they described phenomenon to find characteristics in the narratives; This means that participants’ expressions were translated into a psychological meaning
4) Situated contexture	Each interview text was summarized into a synopsis; the 14 synopses were based on MUs that had gone through eidetic induction into a new “whole” to describe phenomenon as a lived experience and what phenomenon is
5) Results in general characteristics and typologies	First, a general structure and general characteristics were searched for by comparing and identifying similarities among 14 synopses. General structure describes *what* the phenomenon is, whereas general characteristics are essentialities commonly expressed throughout the narratives; Second, similarities were synthesized into general characteristics and formed 3 typologies representing variations in how phenomenon was described in data
Epoché	Epoché means to adopt a reflective and critical attitude and bridle preunderstanding and set aside assumptions and beliefs about phenomenon in focus; epoché was a crucial part of every step of analysis

As noted in step 5, the variations of the phenomenon were interpreted in terms of typological structures. Typological structures are meanings of the phenomenon described by the participants in relation to the aim. Typological structures are considered essential to understanding the “how” in how the phenomenon was experienced and describe the different ways in which the phenomenon appeared in the data. Typologies represent the variations in how the phenomenon is experienced in order to understand the essence of the phenomenon. The variation can be found within an individual’s expression, meaning that 1 participant can be represented in more than 1 typology*.* The first and last authors discussed how the variations in the descriptions by the participants could be understood and formed the 3 typologies. Typologies can be understood in relation to other qualitative methods as major themes. The last author with many years of experience with the EPP method drafted the text for the 3 typologies. The first and last authors discussed the 3 drafted typologies on several occasions, and whenever uncertainties occurred, the first and last authors went back to the original transcribed interviews to confirm the interpretation of the text, as done in step 4.

### Establishing Trustworthiness

Lincoln and Guba have described 4 aspects of trustworthiness in qualitative studies: credibility (confidence in the “truth” of the findings), dependability (refers to the findings being consistent and stable over time and conditions, and also considers the quality of the researcher and the training of the interviewer), confirmability (a degree of neutrality or the extent to which the findings of a study are shaped by the study participants and not researcher bias, motivation, or interest), and transferability (shows that the findings are applicable in other contexts).^24^

Husserl’s phenomenology starts with reflection or epoché. Epoché means “suspension of judgment” or “withholding of assent” and is important regarding credibility. This demands from the researchers a reflective and critical attitude in which one “slows down” the process of understanding to see the phenomenon in a new way.^25^ The researcher, in such an empirical approach, must be as open as possible in relation to the original experience of the phenomenon described by the participants. To bridle the pre-understandings in the researchers performing the analysis (the first and the last author), systematic reflection was used to determine the essential properties and structures of human experience (in relation to SB). The focus was to describe the meaning attributions, as searching for a description of the universal essence, of the phenomenon. This systematic reflection enhances the credibility and also the confirmability of the study.

To further ensure confirmability during the analysis, the first and the last authors independently worked with the material to see if there was a consensus for how the eidetic induction formed the synopsis for each individual interview text. The final typologies were critically reviewed by all authors and contributed to the credibility of the study.

To enhance both dependability and transferability, the research process, the study population, and context where the study took place have been described as accurately as possible without compromising the confidentiality of the participants.

### Role of the Funding Source

The funders played no role in the design, conduct, or reporting of this study.

## Results

A number of general characteristics were found that emerged from the participants’ descriptions of how self-elected and voluntary SB after retirement could be related to health. However, involuntary SB was also perceived as related to ill health. SB was described as a dynamic and changing process to find a balance between being sedentary versus being physically active and performing exercise in everyday life after retirement.

Being sedentary was perceived as a behavior that threatens health and thus something to avoid. Furthermore, being sedentary was also perceived as healthy and existentially meaningful after retirement, and attitudes toward being sedentary had changed for several participants after retirement. A majority of the participants described how they consciously made choices to delay increased SB with routines for activity and exercise after retirement.

**Figure 2:**
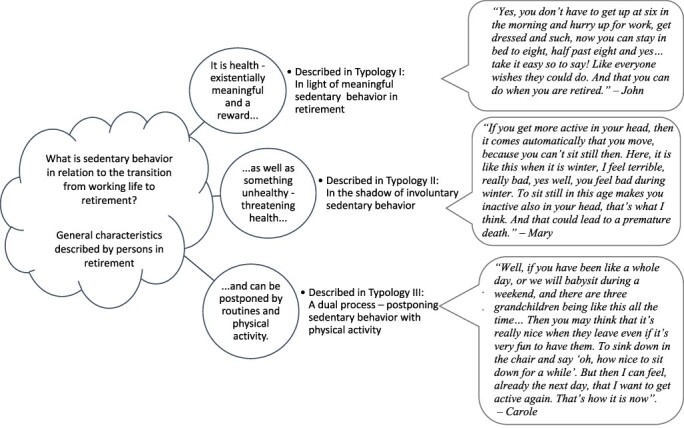
The research question, general characteristics, and the 3 typologies identified illustrated with quotations from the participants.

Three typologies were formed to capture the differences in the descriptions (Fig. 2): in light of meaningful SB; in the shadow of the involuntary SB; and a dual process—postponing SB with physical activity. The typologies are illustrated by quotations by the participants.

### Typology I: In Light of Meaningful Sedentary Behavior in Retirement

The participants describe that being sedentary gained a new meaning after retirement. Retirement was comforting for the body; it was a time for rest, recovery, and a slower pace in life, which contributed to increased awareness of harmony and pleasure. Beliefs were expressed regarding the aging body and how it should be handled. Life was described as good during retirement and was viewed as full-time vacation. The participants expressed an acceptance of the increased need for sedentary time over time as a natural process of aging. They expressed that the body and the soul deserve rest and recovery in retirement:

Yes (answering the question about becoming retired), that’s what I think. It was a full-time holiday, you could say. And you can do things in your own pace, everything you did before. Earlier you may have done 5 things every day. You don’t have to do that today. Today, you do 2 things maybe, that you find important. **–** John

Participants who have had a physically demanding occupation described how the aging body was tired. They expressed an increase in pain, aches, or joint wear and that an older body that carries pains and ailments also needs more rest. SB can thus be understood as a form of escape or a moment’s sanctuary from pain or a lack of energy. The reaction towards more SB may derive from the body’s signals, driving attention to the needs for rest and recovery and to alleviate bodily symptoms that have become increasingly common in retirement. The body was described as more vulnerable to long-term sedentary time than it had been earlier in life as well as more vulnerable to excessive exercise or activity. When aging, physiological signals and bodily vulnerabilities (eg, energy shortages and decreased fitness) are more clearly signaled, and it was described as important to listen to the different signals. Furthermore, this reactive process may also go in the opposite direction, as prolonged sedentary time was also interrupted by restlessness and increased stiffness or more pain and aches. Thus, it was difficult to be sedentary for too long at a time. One needs to move to maintain body circulation or to avoid falling asleep.

The participants described a positive aspect in their freedom to choose everyday activities that they found important or enjoyable, often activities that there had been no time for earlier in life, which included sedentary activities such as playing bridge, having coffee, and photo editing. This self-elected and voluntary sedentary time was described as meaningful and gave instant pleasure but was also described as un-reflected and that it just happened. Several activities performed while sitting are described by the participants as fun or intellectually stimulating, such as solving crosswords, reading an exciting book, or having time for reflection, and were related to health.

Usually, I read, solve crosswords, do sudoku. And my husband and I listen to audiobooks together. Watching TV. And I sew some. I don’t know if that’s what you call sedentary? […] It feels good many times. […] Yes, it is really relaxing and nice to do it [being sedentary]. […] When I am sitting and it feels good, and I can enjoy it. […] I think that I am worth it, to sit during that moment I am sitting. **–**Anna

Within this typology, however, the participants expressed an awareness of the importance of supplementing sedentary time with physical activity. This knowledge of the health benefits of movement was described in relation to the physiological gains of physical exercise. Healthy sedentary time is experienced in relation to being at rest after physical activity, which is good for the aging body. Above all, the body needs to be activated to successfully perform everyday chores or reduce stiffness and pain. The participants described that there are often things to do, such as taking care of a home and house. Furthermore, they described family and friends as playing an important role in naturally decreasing sedentary time, even if many social events are sedentary in nature. The choice of activity was often made based on seasonal activities, such as playing golf during the summer. It was emphasized that it was easier to be physically active during summer, while during winter, it was easier to become more sedentary.

### Typology II: In the Shadow of Involuntary Sedentary Behavior

Within this typology, the participants described how SB was related to times in life in which the participants struggled for some reason. Unhealthy SB was described as an imbalance in relation to physical activity that led to poor health.

Loneliness and social isolation contributed to SB and was also described as leading to withdrawal from physical activities. Loneliness was described as making it more difficult to find activities to engage in, both when active and when sedentary. SB was described to increase with reduced zest for life and boredom. An experience of being mentally out of balance occurred with prolonged and unhealthy SB, especially while in front of the computer or TV.

Being sedentary in solitude was described as a meaningless life. Being alone and sedentary, with a low level of intellectual stimuli, was described as threatening to one’s well-being and health. Being lonely with no planned activities could also lead to anxiety. Participants in this typology described a longing to find like-minded people with whom they could socialize. Socialization could contribute to decreased SB and loneliness. In parallel, the participants described a longing for support to be more active and involved, especially in an accepting social community, having family or friends to visit or routines that included physical activity at home, because environmental distractions play a major role in interrupting SB. Involuntary SB was associated with ill health and was described as something that increased in periods after retirement. Reduced energy and a lack of strength caused by illness or injury could lead to a lack of motivation for physical activity, thus leading to SB, more pain and stiffness, and less energy. Furthermore, the aging body was perceived as more vulnerable to strain. Unforeseen events, such as injuries or recurring illnesses, interrupted the former routines of daily living and created new patterns in life with undesired SB. Not having the physical ability or not being able to do things in the same way as before was frustrating.

Then, I got some problems with a knee which made me quit [certain activities], and then there is some problems like not being able to move as much. And then I got into another pattern in life. I thought it was very disturbing. To not be able to walk properly and such, and it hurt more when one walked and… and… it was kind of disturbing. **–**Peter

SB can be understood as a result of loneliness and negative emotional triggers such as reduced zest for life, which was described as contributing to unhealthy sedentary time. This kind of SB can also result in a negative spiral, leading to more SB. SB itself was also described as a risk factor for being more sedentary. Unhealthy SB was described as not having something to do or waiting for something, such as a way of killing time, unreflective and dull SB, or a quiescent SB, which may have arisen or escalated in retirement. Sometimes SB was also a result of being too comfortable or not having had the character or strength to start again with physical exercise after injury. A fear avoidance of physical activity was described in terms of uncertainty of what was allowed or good to do following physical injury. SB was furthermore described as a reflection of one’s former identity in life. To not consider oneself a sporty person or to not engage in or enjoy physical exercise earlier in life can be understood as maintaining SB after retirement.

I have never been some kind of sporty person. Never. I don’t even like sports on TV. But I don’t want to be any kind of hibernated jock who absolutely has to go to a gym. I don’t go to a gym whatsoever because it smells terrible. I have tried a couple of times but it smells bad. I don’t want to do that. **–**Mary

Trying new activities, even if one wanted to, was described as difficult without social support but also with respect to various personal factors, for example, a lack of enjoyment of physical exercise or illness or injury. If support was available, it was considered to facilitate greater success in reducing SB. Without support in breaking the patterns of SB over time, success might become problematic. It was described as frightening to develop a life pattern in which SB increased, because it was considered to threaten well-being in retirement by creating feelings of meaninglessness, lethargy or tiredness, and killing time with nothing to do as well as to contribute to more bodily pain and stiffness and decreased physical status. Everyday routines and a supportive social environment were described as central to disrupting patterns of unhealthy extended SB (quotation II.C).

Yes, I live a very sedentary life. And it makes it boring. I would like to go out more, I would above all want to have more friends that I could call because I feel anxiety when I am sitting here, and nothing happens. **–**Mary

### Typology III: A Dual Process–Postponing Sedentary Behavior With Physical Activity

The participants in this typology described how SB was nothing that they longed for in retirement and not something of value in itself. In contrast, good health, physical activity, and everyday life activities were the main areas of focus, giving meaning to life in retirement. It was described as important to plan projects and activities so that the body remained physically active throughout the day in relation to SB. Having routines was expressed as critical in interrupting sedentary time. The positive attitude the participants had regarding physical activity before retirement and earlier in life affected SB in retirement. Persons engaged in regular physical exercise or who were active in everyday life before retirement brought that behavior with them into retirement. In that way, the participants described that life continues the way it did before. It was perceived as important not to slow down too much in retirement and to maintain routines for physical activity and exercise. A structured daily life with things to do while physically active was desired. Sedentary time was described as something that naturally interrupted everyday life activities and recovery after physical exercise. In this sense, being sedentary was perceived as valuable and meaningful. Being active led to a need for (healthy) sedentary time.

Participants who had a sedentary working life tended to describe physical exercise, for example, at a gym, as important to staying healthy in retirement. A desire and a longing for physical activity after retirement was described. During periods with less physical exercise, such as after injuries or illnesses, the risk was to slip out of this regularity and become more (unhealthily) sedentary. When events in life interrupted physical activities and exercise, it was expressed as important to make active and conscious choices, have a positive attitude toward exercise, and have a supportive social network to get back to routines.

The physiological benefits of exercise were expressed as an experience of feeling good, increased body flexibility, and improved mental status. The participants described that during periods of no physical exercise, physical health decreased and joint stiffened as a result. Also, other bodily changes associated with poor health became more evident.

And I take walks. And I go to Friskis and Svettis [gym], at least during the half-year winter season and attend 2 classes a week. And that is something I started with when I turned 65. […] and I started lifting weights in the gym because it was really good to go to the gym when you were retired since you could go there before lunch when there was not a queue for the equipment. And I pretty much enjoyed going to the gym, and it was two, three, at least 2 times a week […]. So, it’s that and to walk, that’s what I chose. –Karen

Routines for physical exercise were described as essential for maintaining physical exercise and a natural part of everyday life. Retirement was described as an opportunity to decrease SB. The participants strived to have a meaningful life that included physical activity after retirement (quotation III.B).

The dog, we take him for a walk every morning. We are a whole gang, and dogs together. […]. We are out for an hour. Then you come home and you make breakfast, after breakfast you do this […]. Then, I work in the garden and lite so and so, and I move the lawn and […]. Then, I take the dog for a walk an hour, one and a half maybe. And then it is afternoon…**–**Carole

There were few thoughts or major concerns about the future, but the participants expressed an awareness that involuntary SB may increase when aging, but the participants were not ready to become sedentary yet. The participants in this typology described themselves as very active in everyday life and had many plans. The participants described how SB could be delayed in retirement through various projects, such as renovating the home, helping relatives with various tasks, and exercising. Additionally, retirement was described as a phase in life when one can make new choices for a more active life (quotation III.C).

No, I can’t say that [about being sedentary], I am far from that, depending on what you put in to being sedentary […]. So, it may be that I have difficulties sitting still […]. I set the alarm to half past 7, otherwise I would not get up, […]. And then I do a stretch program that I do every morning, so to speak. And then I eat porridge and read the paper and take a cup of coffee, so let’s say an hour there. Then, I work [garden or renovating]. Then, I go out and do something or… and then I eat lunch… **–**Henry

## Discussion

The objective of this study was to gain a deeper understanding of SB in relation to the transition from working life to retirement as experienced by persons in retirement. The phenomenon of SB can be understood in terms of a shifting duality as related to both health and ill health. SB was, to some participants, related to health and was mostly longed for (typology I). In contrast, there were also participants who described SB as related to ill health (typology II). In typology III, the participants expressed how reflective and conscious choices and daily routines, including physical activity and exercise, may delay SB, which was perceived as a natural process when aging.

**Figure 3:**
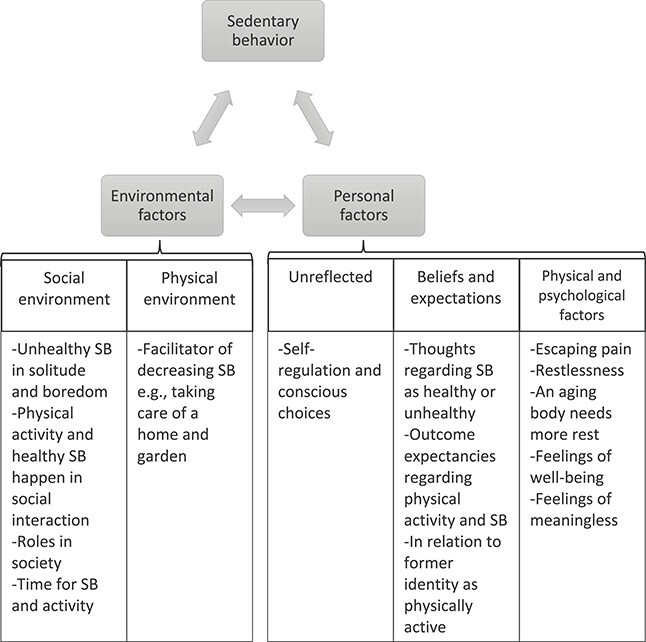
Environmental and personal factors described by the participants related to SCT as influencing the process to and from a sedentary behavior (SB).

When attempting to understand a phenomenon such as SB, it can be advantageous to explain the process to and from the behavior. Thus, the results in this study can be interpreted in relation to social cognitive theory (SCT).^26^ For the understanding of human social behavior, SCT links personal factors, behavior, and environment together in a reciprocal interaction.^26^ The participants described the process to and from SB in terms of a number of personal (physical and psychological) and environmental (social and physical) factors (Fig. 3). Both positive and negative emotions and feelings as well as beliefs about SB as healthy or unhealthy can lead to SB as well as to physical activity. The same goes for environmental factors that can both lead to and from SB. The key in understanding SB is in what context the behavior occurs and also how the individual perceives the behavior in that context as leading to feelings of well-being or the opposite.

The participants in typology I expressed how SB can be a way of escaping from the personal factor of pain, but in a reciprocal manner SB could also lead to bodily signals such as pain, restlessness, and stiffness. Other studies have shown that persons with long-term pain have described that physical activities can mediate pain reduction and be a distraction from it.^27–29^ This was also described by the participants in present study, as SB was also interrupted by restlessness and other bodily signals. Furthermore, long-term pain is a risk factor for frailty in older adults.^30^ Frailty in older adults should be prevented, and by decreasing SB, the risk for developing frailty may also be reduced because there is an association between SB and frailty.^31^ During periods of solitude or sedentary time, inner bodily attention in some patients with long-term pain is often directed towards the problematic body and self^29^ connecting the social environment and personal factors to the behavior. Solitude and SB may thus lead to a negative spiral to more SB due to reciprocal effects on each other. Previous studies have shown an association between depression and SB.^32^ In the present study, the participants expressed that being sedentary in solitude threatened well-being and health. The mental health of older adults is an important aspect of prolonged independent living, and its association with SB is important to consider in interventions aiming to decrease SB. Thus, SB affects individual factors such as feelings of well-being; these factors are also affected by environmental factors (such as loneliness) in a reciprocal manner. Another important aspect expressed by the participants in the present study is also beliefs of how SB is meaningful in retirement and something that gives a feeling of well-being. The participants expressed that there is time for and expectations of being able to be more sedentary and give rest to the aging body when in retirement.

A systematic review and meta-synthesis on how older adults perceived the acceptability of physical activity in aging found that the perception of self-identity and roles within the wider society affected the level of physical activity when aging.^15^ Those who engaged in physical activity in older age did so because it had formed part of their identity earlier in life.^15^ This confirms the findings in typology III in the present study. Beliefs regarding the benefits and appropriateness of being sedentary or physically active and its relationship to (ill) health influence SB and whether individuals are physically active. Furthermore, self-identity has been shown to influence how a sedentary lifestyle is more permissible and accepted by aging persons and is something that feels good,^15^ confirming the findings in typology I and explaining how the social environment can both encourage physical activity and promote SB in older adults by expecting that. The findings in the systematic review^15^ further confirm the findings in the present study in typology II regarding how the aging body is perceived to have limitations that inhibit physical activity and how fear of injury and avoidance beliefs when in pain were barriers to physical activity. It has been suggested that the approach of integrating functional exercise into daily life, rather than structured exercise programs, is a favorable option to reduce SB in older adults.^33^ Reducing SB by increasing functional activities can be more easily implemented because they are closely aligned with the accomplishment of desired daily tasks.^33^

The transition to retirement is a major life event and can be a critical window for interventions to improve health.^34^ This was described by participants who created everyday routines for physical activity in retirement. The participants described the importance of paying attention to their new choices in everyday life to reflect on what leads to SB and what takes one out of it, which can be related to the concept of self-regulation in SCT.^26^ One of the most important findings in this study was that SB often occurred due to unreflective and conscious choices, and being able to self-regulate^35^ was described as a key factor in decreasing sedentary time. To be able to self-regulate and develop new healthy behaviors, one must be knowledgeable of the risks of a behavior.

In a clinical setting, it is important for the physical therapist to identify how clients/patients perceive or experience their everyday life in relation to SB in order to support clients’/patients’ health-related behavior changes. For example, when a physical therapist meets an older patient with chronic low back pain, it is important to discuss how the patient sees themselves as being sedentary when the back pain limits their activities. Positive and/or negative narratives of SB should be encountered. If a patient describes that their activities in everyday life are restricted due to the chronic low back pain leading to an involuntary SB, it is important that the physical therapist can identify the involuntary SB. If the patient performs activities sitting due to pain that may be possible to perform while standing, the physical therapist can contribute with insights regarding the importance to vary the position and reducing sedentary time by making small changes in actives in everyday life. Together with the patient, the physical therapist, with their unique competences regarding pain and activities, may find alternative positions to vary sitting and standing and thus support the patient to reduce the involuntary sedentary time.

Because actions taken to support behavior change are related to the understanding of a complex behavior (ie, exercise behavior), we first need to describe and understand the phenomenon from a life-world perspective^18^ to support individuals to change it.^36^ According to the results in the present study, the identification regarding if the SB is voluntary or not is of significance because clients seem to relate this to health and well-being. When physical therapists encounter persons in the transition from working life to retirement, SB should be addressed. Information about SB and a possible negative effect on health should be offered. In the present study, the environment (both social and physical as well as the inner motivation and personal beliefs, etc) was reported as important to decrease or increase SB. Physical therapists can support the clients/patients to transform the environment to give opportunities for decreasing the unhealthy SB and to find a balance between healthy SB and activity. Physical therapists can encourage clients/patients to seek social support for activity in their everyday life or transform the clients’/patients’ physical environment, for example, advising the client/patients to procure a height-adjustable table to enable the patients/clients to perform task usually performed sitting in standing. This might be even more important during colder seasons when SB is more common according to present study.

### Limitations

The results did not reveal a general structure due to the variations of the descriptions among the participants. This is not uncommon in phenomenological studies (see^27^ as an example), especially in regard to a complex phenomenon such as SB, which can be experienced in multiple ways.

There was a large range in the time since retirement (1–13 years) among the participants. It is possible that the large range in time since retirement may have influenced the results, particularly regarding loneliness and establishing routines in everyday life. Longer time since retirement may increase loneliness and lack of routines. This study may not have succeeded in recruiting persons in the immediate transition from working life to retirement, which may therefore be of interest in future studies.

The snowball sampling used may have resulted in participants recruiting other participants similar to themselves. Nevertheless, the participants differed regarding former occupations and living conditions. It has been shown that there is a difference in physical activity levels between lower and higher socioeconomic groups in retirement.^37^ In the present study, participants who described heavier work tasks before retirement expressed the need for recovery and to escape from bodily pain and therefore welcomed SB compared with persons with mostly desk work. The type of work could be considered to be related to socioeconomic status, but socioeconomic status alone may not explain differences in SB in retirement among different socioeconomic groups. Earlier studies have had low representation of men,^10–12^^,^^15^ and sex heterogeneity is a strength in the present study. However, all participants were ethnic Swedes who contributed to a homogenic sample in that aspect, which could be a limitation regarding transferability of the study results to other groups. This study was conducted in the middle of Sweden where the climate differs with the season: cold winters with snow and heat during the summer. The results in our study related to participants’ description of SB may therefore not be transferable to populations of older adults living in another climate zone.

In a phenomenological approach, the researcher must be as open as possible in relation to the original experience of the phenomenon as described by the participants.^38^ This includes ensuring credibility by moving back and forth between the interpretations and the data by letting the researcher reflect and refine the dimensions of the overall meaning structure.^23^ To bridle the preunderstanding of the authors involved in the data analysis, the preunderstanding was continually discussed and reflected on, strengthening the credibility of the present study. Member checks were not performed in the present study, which might be a limitation. Member check has been argued as a crucial method to improve trustworthiness of qualitative studies.^24^ The member check process typically includes that the participants receive a copy of the findings and annotated themes with the opportunity to reflect and comment on the findings based on their own experiences.^39^ But there is also critique towards member check as a method for improving trustworthiness in that sense that it can be difficult for the participants to follow because the results have been synthesized, decontextualized, and abstracted from and across individual participants to the extent that it might be difficult to recognize themselves or their own experience.^40^ This might be even more evident in a phenomenological study using the EPP because the level of interpretation to a higher psychological meaning can be even more difficult to follow; this was why member checks were not used in the present study. In this study, the systematic reflections to bridle the preunderstanding, the first and the last author independently working with the analysis as well as the final typologies being discussed within the author group strengthen the trustworthiness of the results.

The experienced meaning of SB in retirement can be a key in developing an understanding of health and ill health among adults after the transition from working life to retirement. Additionally, a deeper understanding of the meaning of SB in this group may inform interventions for reducing SB to be more acceptable. This study fills a gap regarding what SB means in retirement, which may be helpful in targeting those in need of support in decreasing SB and individualizing interventions to decrease SB for persons in retirement. Future research should focus on how to encourage decreased SB among those in transition from working life to retirement.
